# The level of kinesiophobia in breast cancer women undergoing surgical treatment

**DOI:** 10.3389/fonc.2023.1010315

**Published:** 2023-02-02

**Authors:** Ewa Malchrowicz-Mośko, Piotr Nowaczyk, Janusz Wasiewicz, Tomasz Urbaniak, Wojciech Siejak, Mateusz Rozmiarek, Urszula Czerniak, Anna Demuth, Aitor Martinez Aguirre-Betolaza, Arkaitz Castañeda-Babarro

**Affiliations:** ^1^ Department of Kinesiology, Faculty of Sport Sciences, Poznan University of Physical Education, Poznan, Poland; ^2^ Breast Surgical Oncology Department, Breast Cancer Unit, Greater Poland Cancer Center, Poznan, Poland; ^3^ Faculty of Education and Sport, University of Deusto, Bilbao, Spain

**Keywords:** female oncology, fatigue, hypokinesia, fear of movement, cancer

## Abstract

**Introduction:**

Lifestyle-associated factors like physical activity (PA) play an important role in cancer prevention and oncology treatment outcomes. The aim of the study is to investigate the level of kinesiophobia (fear of movement) in breast cancer (BC) patients undergoing surgical treatment depending on socio-demographic variables, lifestyle before cancer diagnosis, stage and type of BC and comorbidities.

**Methods:**

We interviewed 285 women (132 patients from Greater Poland Cancer Center – age: 55.7 ± 12.4; BMI: 26.7 ± 4.7 and 153 healthy women from control group – age: 49.0 ± 15.7; BMI: 25.7 ± 4.0) using Polish adaptation of the Tampa Scale of Kinesiophobia (TSK).

**Results:**

Research results show that women with BC suffer from kinesiophobia (>37 points) signi!cantly. Approximately 3/4 of the surveyed women with BC did not know the World Health Organization (WHO) recommendations regarding the weekly dose of PA for healthy people and for people with cancer. Before cancer diagnosis more than a half of women (60%) performed PA in accordance with WHO recommendations. 7% less women performed PA during oncology treatment. Almost a half of patients are not physically active during cancer treatment and 1/5 of the respondents declared that they do not know yet if they will be physically active after oncology treatment. The level of kinesiophobia in BC women with comorbidities was the same as in the group of BC women without comorbidities. However, the highest levels of fear of movement have been observed among women with BC suffering also from osteoporosis, obesity and diabetes. In general, higher levels of kinesiophobia were reported among women in less advanced stages of the disease. There were no differences in the level of kinesiophobia depending on the type of BC (hormonally dependent luminal cancers vs. other types). The level of kinesiophobia did not differ between women who were physically active before BC diagnosis and women who were not. In terms of socio- demographic variables, we found one direct association between the level of kinesiophobia (pain) with age – the greater age, the higher level of pain kinesiophobia.

**Discussion:**

Research on fear of movement in female oncology requires further research (including also chemotherapy, radiotherapy, immunotherapy and hormonal therapy) and in order to effectively eliminate hypokinetic attitudes at every stage of cancer treatment.

## 1 Introduction

Lifestyle-associated factors play a significant role in cancer prevention. There is strong evidence that physical activity (PA) before, during, and after diagnosis improves outcomes for breast cancer (BC). The findings suggest that PA protects against recurrence and progression in BC survivors (1). In addition to hereditary and environmental factors, modifiable factors such as lifestyle, including insufficient level of PA and poor and improper diet, also affect the incidence of BC and its recurrence. It is estimated that 9 in 10 cases of BC are due to non-genetic factors, and approximately 25% to 30% of total BC cases should be preventable only by lifestyle interventions. BC are also attributable to reproductive factors, directly related to hormonal levels and modifiable risk factors like overweight, obesity and sedentarism (2). Obesity plays an important role in particular in the etiology of hormone-dependent (luminal) breast cancers. Whereas ovaries are the most important source of estrogen in premenopausal women, adipose tissue is the main producer in postmenopausal women, having a profound impact in ER-positive (estrogen-positive) BC incidence (3, 4). Overweight and obesity are related with low-grade chronic inflammation, insulin resistance, hyperactivity of cell signaling pathways such as insulin-like growth factor, and adipose tissue dysregulation, which are triggers of breast carcinogenesis (5). Visceral and intramuscular adiposity are also considered potential risk factors of cardiovascular complications after BC diagnosis. Beneficial effects of PA in weight loss, body composition, and immune and metabolic parameters seem to be the most important action of exercise in BC prevention and management (6). Obesity and weight gain are negative prognostic factors for BC survival. PA prevents weight gain and may decrease obesity (7). Studies suggest that many cancer survivors are insufficiently physically active – older, overweight and obese women had significantly lower moderate-to-vigorous PA than their younger male normal-weight counterparts (1). The main physiologic barriers to PA participation among oncology patients are cancer-related or treatment-related side effects (e.g., fatigue) and prevalent comorbidities (8). Regular exercise in BC survivors has been linked to reductions in cancer-related fatigue, nausea symptoms and improvements in immune system function (9, 10). PA may be an effective intervention in reducing state anxiety in BC patients, especially those with high state anxiety (11). PA has been also recognized as a potential intervention to improve quality of life in women with BC (12–14).

In 2020, World Health Organization (WHO) issued official recommendations that adult cancer patients should perform the same weekly dose of PA as fully healthy people (15). Unfortunately, many oncology patients who exercised before the diagnosis do not return to the same exercise level (9). BC survivors are significantly less physically active within their first year after diagnosis than they were one year before diagnosis (7). Moreover, greater decreases in PA after BC diagnosis observed among heavier patients imply a potential for greater weight gain among women who already are overweight (16). PA exerts its greatest benefits to women with BC during or after menopause, or among patients with body compositions (BMI – body mass index, body fat, waist circumference) in the upper-normal-to-overweight range (17).

Fear has been identified as a barrier to exercise for people with neuromusculoskeletal conditions but has been minimally explored in women with BC (9). There is little study in the literature to evaluate the rate of kinesiophobia in BC patients and survivors (18). Kinesiophobia, the fear that movement and PA could worsen side effects such as fatigue and pain, is a barrier to exercise in cancer patients (19). Kinesiophobia negatively affects the level of PA (20). Pain-related fear has been increasingly recognized as an important contributor to the maintenance of (chronic) pain. Following cancer treatment and a prolonged period of rest during oncology treatment, cancer survivors may perceive activity levels that were previously well tolerated as strongly fatiguing. If, due to increased fear levels, one of the responses is to avoid or limit PA levels instead of gradually increasing it, then the fatigue may paradoxically be persisting and leading to a reduced quality of life. Such avoidance has been hypothesized as central to fear of pain and chronic fatigue and is called kinesiophobia. Patients with chronic fatigue syndrome have been shown to be less physically active and have longer rest periods after exercise than healthy people (21–23). Physical inactivity is especially visible in older cancer patients and is associated with a faster decline in functional capacity, an increased mortality risk and a reduced quality of life, even years after the end of cancer treatments (24).

Research evidence indicates that women should return to normal use of their arm after BC surgery. However, it appears some women think that they are supposed to protect their arm from strenuous activities because of the risk of lymphedema (25). Lymphedema is a chronic and progressive long-term adverse effect of BC treatment commonly defined by swelling of the affected arm. Clinical guidelines indicate that women with and at risk for lymphedema should protect the affected arm from overuse. In clinical practice, this often translates into risk aversive guidance to avoid using the arm. This could lead to a disuse pattern that may increase the likelihood of injury from common activities of daily living. Further, such guidance poses an additional barrier to staying physically active, potentially translating to weight gain, which has been shown to be associated with worse clinical course for women with lymphedema (26). It is well-known that BC survivors with decreased PA are at an increased risk for upper extremity lymphedema, and PA and exercise are important to improve the lymphatic drainage *via* the muscles to contract (27). The main barriers to the participation in PA according to previous studies are secondary lymphedema, fear of pain and fatigue, lack of information about the permissible type of activity, bad mood, depression and apathy (28). The results of previous exploratory study conducted by Wong (2014) suggest that the reported daily activity limitations of women who have had BC surgery may be related to the participant pain perception and/or fear of PA (29). The literature shows that a high level of kinesiophobia correlates with a low level of PA (30).

## 2 Materials and methods

### 2.1 Aim of the study

The aim of the study is to investigate the level of kinesiophobia in BC patients undergoing surgical treatment depending on socio-demographic variables, lifestyle before cancer diagnosis, stage and type of BC and comorbidities.

### 2.2 Procedure and research tool

The Tampa Scale for Kinesiophobia (TSK) is one of the most frequently employed measures for assessing fear of movement in patients. The scale was developed in English by Miller et al. in 1991 (31). The TSK has been translated, adapted and validated in many countries, e.g., Japan (32), Portugal (33), Spain (34), Sweden (35), and used in many diseases, e.g., neck pain (36), myocardial infarction (37), multiple sclerosis (38), Parkinson’s disease (39) or rheumatoid arthritis (40).

The TSK contains 17 statements/items in which patients respond according to a four-point Likert scale, ranging from 1 to 4: “I do not agree completely”, “I do not agree in part”, “I agree partially”, “I agree completely”. The scoring of questions 4, 8, 12, and 16 is reversed. The total score is in the range of 17–68 points. The higher the score the greater the severity of kinesiophobia. Researchers developed a cut-off score and reported patients that scored greater than 37 were high-responders (18, 21, 22). Similarly, we used this cut-off score to identify the BC patients with kinesiophobia. In our diagnostic survey, during interviews with oncology patients we used questionnaires with TSK which specifies fatigue (41) and pain (21, 22).

Our goal was to identify the attitudes of BC patients towards PA during surgical treatment depending on socio-demographic variables (age, education, professional situation, marital status, place of residence – rural vs. urban areas), stage of neoplastic disease, type of BC, pre-disease PA and the presence of additional chronic diseases other than cancer. The study used the Polish adaptation of the TSK questionnaire to examine attitudes towards PA among cancer patients (**Appendix 1**) (42). Thanks to this research tool patients have the opportunity to evaluate several dozen statements on a scale which allows them to find out to what extent patients feel fear of movement. The prepared questionnaire consisted of five parts: a) socio-demographic data, b) TSK questions, c) information on the lifestyle (including the weekly level of PA before and during the disease according to WHO), knowledge of the current WHO recommendations, PA plans after treatment, etc., d) stage of the neoplastic disease and type of BC and e) the existence of additional chronic comorbidities (such as obesity or osteoporosis, which may increase the fear of movement). The surgical department for breast diseases at the Greater Poland Cancer Center reported that an average of 800 new cases of BC are treated there each year. We tried to include at least 10% of patients in our study. Doctors explained questions about the level of kinesiophobia and the level of PA to the respondents. The list of questions regarding PA is included in **Appendix 2**. Doctors explained to the respondents what is meant by PA – both recreational PA (in leisure time) and, for example, the way of active transport to the workplace. Both questionnaires were designed in the same way. The control group did not fill in the questions about the course of the neoplastic disease. The collection of anonymous data was done by doctors and their analysis by scientists-statisticians.

### 2.3 Ethical issues

Our study protocol was reviewed and approved by Bioethics Committee at Poznan University of Medical Sciences in Poland (16 June 2021). A decision was issued that the study does not have the features of a medical experiment and that our diagnostic survey may be conducted among cancer patients in the oncological surgery department of breast diseases (and also in a control group of healthy women). The director of the Greater Poland Cancer Center in Poznan and the director of POSUM (Poznański Ośrodek Specjalistycznych Usług Medycznych) in Poznan also gave the written consent for our study (1 June 2021). Both written consents were submitted with the manuscript while submission. The issues of confidentiality and anonymization of data have been agreed with the Greater Poland Cancer Center Personal Data Officer. After talking to patients, the researchers-interviewers threw the questionnaires into one urn. The study was carried out by the method of an anonymous, voluntary diagnostic survey using the TSK questionnaire. The respondents were informed about the goals of the research. The respondents could stop participating in the study at any time. Answering the questions was treated as informed consent to participate in the study.

### 2.4 Statistical analysis

Descriptive data were expressed in % or mean ± SD. The Kolmogorov-Smirnov test was used to evaluate the distribution of normality. The unpaired t-test to compare the demographic and clinical characteristics between the groups were conducted. Cohen´s d values were calculated to assess the effect size. The Pearson’s chi-square test was used to analyze categorical data. The Spearman’s rank correlation coefficients were used to evaluate the relationship between the TSK scores and the rest of the variables. The Spearman’s rank correlation coefficients were accepted as follows: 0.81-1.0 as excellent, 0.61-0.80 very good, 0.41-0.60 good, 0.21-0.40 fair, and 0-0.20 poor. A p value of less than 0.05 was considered statistically significant. Statistical analysis was performed using the IBM SPSS Statistics 28.0 (SPSS Inc., Chicago, IL, USA).

### 2.5 Characteristics of the respondents

The study among 285 women was conducted from September 2021 to March 2022 at the Breast Cancer Unit operating at the Greater Poland Cancer Center in Poznan (interviews with 132 women suffering from BC have been conducted by oncologists – T. Urbaniak, P. Nowaczyk, W. Siejak, Z. Wasiewicz) and at the POSUM (Poznański Ośrodek Specjalistycznych Usług Medycznych) in Poznan (interviews with 153 women without BC have been conducted by the researchers from Poznan University of Physical Education) – control group. Only 18+ women took part in the study. All women admitted to the hospital during the study (Sept. 2021 – March 2022) were invited to participate in the interview by the doctors who participated in this research project. The study in the control group of women without BC did not include women suffering from another type of malignant tumor and who had suffered a stroke, heart attack or spinal cord injury.

## 3 Results

### 3.1 Characteristics of women with BC and women without BC

**Table 1** shows the characteristics of the 285 surveyed women. The mean age of 132 women with BC was slightly higher than that of a control group of 153 healthy women (55.7 vs. 49). The women in both groups were above normal and overweight (BMI: 26.7 and 25,7). The majority of women with BC declared secondary education (51.5%) and the majority of healthy women declared higher education (51.6%). The majority of women with cancer live in rural areas (33.3%) and the majority of healthy women in large cities (50.3%). Both groups are professionally active (47.3% and 56.2%). One in five women is divorced in both the sick and control groups (22% and 19.6%). Healthy women were more often singles. Both groups feel slight fatigue and slight pain mostly. Healthy women feel also slight pain and in BC group women don’t feel any pain at the moment.

**Table 1:** Characteristics of the surveyed women (N=285) – women with BC (N=132) and control group (N=153) – women without BC. Data is shown as mean ± standard deviation (SD) and percentages (%).

Characteristics of the sample	Cancer group N=132	Healthy group N=153
Age (years)	55.7 ± 12.4	49.0 ± 15.7
BMI (kg/m^2^)	26.7 ± 4.7	25.7 ± 4.0
Achieved level of education	Higher: 40.9%	Higher: 51.6%
	Secondary/or status of student: 51.5%	Secondary/or status of student: 37.9%
	Primary: 7.6%	Primary: 10.5%
	Higher: 40.9%	Higher: 51.6%
Place of residence	Village: 33.3%	Village: 18.3%
	Small city (less tan 20 000 inhabitants): 17.4%	Small city (less than 20 000 inhabitants): 7.2%
	Medium city (20-100 000 inhabitants): 25.1%	Medium city (20-100 000 inhabitants): 24.2%
	Large city (more than 100 000 inhabitants): 24.2%	Large city (more than 100 000 inhabitants): 50.3%
	Active: 47.3%	Active: 56.2%
	Pensioner (age reason): 41.2%	Pensioner (age reason): 22.9%
Occupational status	Pensioner (health reason): 4.6%	Pensioner (health reason): 11.8%
	Student: 0%	Student: 5.9%
	Unemployed: 6.9%	Unemployed: 3.2%
	Single: 6.8%	Single: 24.2%
	In marriage/	In marriage/
Marital status	relationship: 70.4%	relationship: 56.2%
	Widow: 0.8%	Widow: 0%
	Divorced: 22%	Divorced: 19.6%
	Slight fatigue: 37.1%	Slight fatigue: 31.4%
Do you feel fatigue?	Medium fatigue: 20.4%	Medium fatigue: 30.7%
	Strong fatigue: 6.1%	Strong fatigue: 15.7%
	I don’t feel fatigue now: 36.4%	I don’t feel fatigue now: 22.2%
	Slight pain: 34.1%	Slight pain: 39.9%
Do you feel pain?	Medium pain: 18.2%	Medium pain: 30.7%
	Strong pain: 1.5%	Strong pain: 15%
	I don’t feel pain now: 46.2%	I don’t feel pain now: 14.4%

BMI, Body Mass Index.

### 3.2 Lifestyle of BC patients

At the time of the study approximately 3/4 of the surveyed women with BC did not know the WHO recommendations regarding the weekly dose of PA for healthy people (73.5%) and for people with cancer (77.1%) (**Table 2**). Before cancer diagnosis more than a half of women (60.6%) performed PA in accordance with WHO recommendations. 7% less women performed PA during treatment (53%). Almost half of patients are not physically active during cancer treatment and 1/5 of the respondents declared that they do not know yet if they will be physically active after oncology treatment.

**Table 2:** Lifestyle of BC patients (N=132).

Characteristics of the sample	Cancer group N=132
Did you know WHO physical activity recommendations for healthy adults?	Yes: 26.5%
	No: 73.5%
Do you know WHO physical activity recommendations for people with cancer?	Yes: 22.9%
	No: 77.1%
Did you lead an active lifestyle before BC diagnosis? (in accordance with WHO recommendations which have been presented by the researchers during interview)	Yes: 60.6% No: 39.4%
For how many years have you been physically active? (in accordance with WHO physical activity recommendations)	12.3 ± 15.7
For how many years you have not been physically active? (not in accordance with WHO physical activity recommendations)	15.2 ± 14.2
Do you lead an active lifestyle now during oncology treatment?	Yes: 53% No: 47%
	Yes: 81.1%
Are you going to lead an active lifestyle after oncology treatment?	No: 0%
	I don’t know yet: 18.9%

BC, breast cancer; WHO, World Health Organization.

### 3.3 The level of kinesiophobia among BC patients

The level of kinesiophobia among women with BC and women without BC (control group) is presented in **Table 3**. Researchers developed a cut-off score and reported patients that scored greater than 37 were high-responders (18, 21, 22). Similarly, we used this cut-off score to identify the BC patients with kinesiophobia. Both groups showed that they felt the phenomenon of kinesiophobia. It concerns both the fear of movement due to the expected pain and the expected fatigue. There were no significant differences between the groups neither in fatigue (P=0.220) nor in pain (P=0.127).

**Table 3:** Kinesiophobia rates of each group.

Average Kinesiophobia	Cancer group N=132	Healthy group N=153	p	Cohen’s d
Fatigue	40.8 ± 7.6	40.1 ± 6.3	0.220	6.989
Pain	41.9 ± 7.5	40.9 ± 6.6	0.127	7.057

Data is shown as mean ± standard deviation (SD). Statistical significance set at P<0.05.

### 3.4 The level of kinesiophobia and lifestyle before BC diagnosis

The comparison of kinesiophobia during treatment (N=132) among women who were previously physically active (before cancer diagnosis) and women who were not physically active is presented in **Table 4**. It turned out that the level of kinesiophobia did not differ statistically significantly between women who were active and women who were not (fatigue P=0.101; pain P=0.213). The previous lifestyle does not influence on the level of kinesiophobia during cancer treatment.

**Table 4:** Level of kinesiophobia and lifestyle – the impact of lifestyle before diagnosis on the level of kinesiophobia during cancer treatment.

Average Kinesiophobia	Women physically active before treatment N=80	Women not physicall active before treatment N=52	p	Cohen’s d
Fatigue	40.1 ± 8.6	41.9 ± 5.9	0.101	7.627
Pain	41.4 ± 8.5	42.5 ± 5.8	0.213	7.550

Data is shown as mean ± standard deviation (SD). Statistical significance set at P<0.05.

### 3.5 The level of kinesiophobia according to cancer stage (pre-invasive & early stage vs. late BC)

Statistically significant higher levels of kinesiophobia were observed among women in less advanced stages of the disease (P=0.042) (**Table 5**).

**Table 5:** Level of kinesiophobia and cancer stage.

Average Kinesiophobia	Pre-invasive or early BC (stages: 0,1,2)	Late BC (stages: 3,4)	p	Cohen’s d
Fatigue	40.4 ± 7.2	39.0 ± 8.7	0.063	7.606
Pain	42.5 ± 7.1	39.8 ± 8.5	0.042*	7.482

BC, breast cancer.

Data is shown as mean ± standard deviation (SD). Statistical significance set at P<0.05. *statistically significant.

### 3.6 The level of kinesiophobia according to BC type (Luminal A & B vs. HER2+ vs. Basal/Triple negative)

Endogenous estrogens signaling have been shown to play a central role in the initiation and development of BC particularly in postmenopausal women (43). PA is a negative modulator of estrogen levels which aid to explain the preventive role of PA in women after menopause (44, 45). PA plays a particularly important role in the development of hormonally dependent BC. The level of kinesiophobia among women with hormonally dependent luminal cancers is presented in **Table 6**. There were no statistically significant differences in the level of kinesiophobia depending on the type of BC (fatigue P=0.296; pain P=0.131).

**Table 6:** Level of kinesiophobia and type of BC.

Average Kinesiophobia	Luminal (A+B)	HER2+	Basal/Triple negative	p	F value	Effect size
Fatigue	41.0 ± 8.1	39.6 ± 5.7	43.1 ± 9.1	0.296	1.228	0.019
Pain	42.1 ± 8.1	40.2 ± 5.7	44.7 ± 8.1	0.131	2.064	0.031

Data is shown as mean ± standard deviation (SD). Statistical significance set at P<0.05.

### 3.7 The level of kinesiophobia according to comorbidities

The main physiologic barriers to PA participation among oncology patients are cancer-related or treatment-related side effects (e.g., fatigue) and prevalent comorbidities (8). The level of kinesiophobia among people with and without comorbidities is presented in **Table 7**. The level of kinesiophobia in both groups of the respondents resulted similar with no statistical differences between them (fatigue P=0.247; pain P=0.375). Then the level of fear of movement in every surveyed chronic disease was checked (**Table 8**). The highest levels of kinesiophobia have been observed among women with osteoporosis, obesity and diabetes.

**Table 7:** Level of Kinesiophobia according to other comorbidities.

Average Kinesiophobia	Without other comorbidities	With other comorbidities	p	Cohen’s d
Fatigue	41.3 ± 7.8	40.4 ± 7.5	0.247	7.661
Pain	42.1± 7.7	41.7 ± 7.5	0.375	7.566

Data is shown as mean ± standard deviation (SD). Statistical significance set at P<0.05.

**Table 8:** Level of kinesiophobia and comorbidities.

	Average Kinesiophobia Fatigue	P	Cohen’s d	Average Kinesiophobia Pain	P	Cohen’s d
Without diabetes	40.7 ± 7.6	0.162	7.646	41.7 ± 7.5	0.124	7.530
With diabetes	**43.8** ± 8.0			**45.3** ± 8.4		
Without hypertension	41.3 ± 7.7	0.186	7.652	42.3 ± 7.6	0.209	7.550
With hypertension	40.1 ± 7.5			41.2 ± 7.5		
Without obesity	40.6 ± 7.9	0.178	7.650	41.5 ± 7.7	0.104	7.523
With obesity	42.4 ± 5.9			**44.0** ± 6.2		
Without osteoporosis	40.7 ± 7.3	0.325	7.649	41.7 ± 7.2	0.351	7.549
With osteoporosis	**44.0** ± 15.1			**44.6** ± 15.5		
Without arteriosclerosis	40.9 ± 7.7	0.270	7.664	41.9± 7.6	0.474	7.569
With arteriosclerosis	37.5 ± 0.1			41.5 ± 3.5		

Data is shown as mean ± standard deviation (SD). Statistical significance set at P<0.05.

### 3.8 The level of kinesiophobia according to socio-demographic variables

We found one direct association between the level of kinesiophobia (pain) with age (P=0.016). It means that the greater age, the higher level of pain kinesiophobia. No associations were found in the other socio-demographic variables (**Table 9**).

**Table 9:** Correlation of Kinesiophobia scores with sociodemographic variables; r: The Spearman’s rank correlation coefficients.

	Kinesiophobia fatigue		Kinesiophobia pain	
	r	P	r	P
Level of education	-0.018	0.757	-0.027	0.655
Place of residence	0.034	0.568	-0.033	0.578
Marital status	-0.026	0.663	-0.053	0.375
Occupational status	-0.052	0.385	-0.059	0.320
Age	-0.095	0.109	0.142	0.016*

They were accepted as follows: 0.81-1.0 as excellent, 0.61-0.80 very good, 0.41-0.60 good, 0.21-0.40 fair, and 0-0.20 poor. A P value of less than 0.05 was considered statistically significant. *statistically significant.

## 4 Discussion

Previous study showed that oncology patients who were physically active before BC diagnosis are not afraid to exercise. In our study, BC patients who were physically active before diagnosis have the same level of kinesiophobia as women who were not active before oncology treatment. The level of kinesiophobia in patients with BC in Turkey was found to be 30.8%. The level of kinesiophobia in Turkish BC patients with lymphedema was significantly higher than those without lymphedema (18). In our study, the level of kinesiophobia among BC patients in Poland is much higher (+10-12%). It may result from less effective PA management and education in oncology hospitals in Poland or it could have been influenced by the coronavirus pandemic.

Approximately 3/4 of the surveyed women with BC did not know the WHO recommendations regarding the weekly dose of PA for healthy people and for people with cancer. Before cancer diagnosis more than a half of women (60%) performed PA in accordance with WHO recommendations. 7% less women performed PA during treatment. Almost half of patients are not physically active during cancer treatment and 1/5 of the respondents declared that they do not know yet if they will be physically active after oncology treatment. In general, the BC patients reported quite positive attitudes towards PA, so they probably recognize the benefits of PA for mental and physical well-being and quality of life. However, the results of the research showed that the level of PA of the surveyed women and their knowledge of the WHO recommendations are still insufficient and only half of them is physically active during BC treatment.

Statistically significant higher levels of kinesiophobia were reported among women in less advanced stages of the disease (P=0.042). Perhaps this is due to the popular and wrong belief that physical movement can cause cancer cells to spread and reveal cancer metastasis. As women with early BC have a good prognosis and PA plays an important role in prevention, special efforts should be made to eliminate the phenomenon of kinesiophobia in this group of patients.

PA plays a particularly important role in the development of hormonally dependent breast cancers. In our study there were no statistically significant differences in the level of kinesiophobia depending on the type of BC (hormonally dependent luminal cancers vs. other types) (fatigue P=0.296; pain P=0.131). However, women with hormonally dependent breast cancers should have lower levels of kinesiophobia in order to be physically active as often as possible. There is evidence for an inverse association between PA and BC risk and evidence is stronger especially for postmenopausal BC (than for premenopausal BC) (46). Research results suggest that moderate PA, including brisk walking, may reduce postmenopausal BC risk and that increases in PA after menopause may be beneficial (47). Low levels of PA in women of perimenopausal age favour kinesiophobic attitudes and thereby increase the level of barriers against undertaking PA (48).

The BC treatment often involves hormonal therapy, the side effect of which may be osteoporosis which can lead to fractures. PA is essential preventive and therapeutic approach for osteoporosis. Having a diagnosis of osteoporosis without an adequate education about the disease may lead to kinesiophobia in patients due to an illogical belief about increasing possibility of falls and related fractures during PA. According to Gunendi et al. (2018), people with osteoporosis had higher level of kinesiophobia than healthy control subjects (49). BC and cardiovascular disease are conditions directly correlated (1). BC survivors are becoming increasingly predisposed to cardiovascular disease mortality. Low cardiorespiratory fitness and PA levels, as well as high values of adiposity indices, contribute to cardiovascular disease risk (50). In our study, the highest levels of kinesiophobia have been observed among women with osteoporosis, obesity and diabetes. Regular PA is extremely important in these chronic diseases. Women with abnormal body weight show a fear of movement because in their opinion they tire quickly and may have difficulty performing certain body movements, women with osteoporosis are probably afraid of falls and injuries and women with diabetes may experience the effects of diabetic polyneuropathy, among other things. Increased body weight at the time patient is diagnosed with BC is associated with an increased risk of recurrence and reduced survival. Weight gain is also common after diagnosis. Increasing PA after diagnosis may minimize these adverse outcomes. Unfortunately, according to Irwin et al. (2003), patients with BC decrease their total PA from pre-diagnosis to postdiagnosis and greater decreases in PA are observed among obese patients compared with patients of normal weight (16). The study by Invernizzi et al. (2022) showed that regular PA therapy in patients with BC showed significant results in terms of metabolic biomarkers, including glycemic, insulin and lipid profiles (51). Moreover, the protective effects of PA on cancer risk are hypothesized to be through multiple interrelated pathways: decrease in adiposity, decrease in sexual and metabolic hormones, changes in biomarkers and insulin resistance, improvement of immune function, and reduction of inflammation (52). Anti-cancer therapies lead to chronic non-resolving inflammation and reduced immune function. One potential therapy is PA which reduces pro-inflammatory markers in cancer survivors, with the strongest evidence for combined training and for breast and prostate cancer survivors (53).

In terms of socio-demographic variables, we found one direct association between the level of kinesiophobia (pain) with age (P=0.016; r=0.142). It means that the greater age, the higher level of pain kinesiophobia. No associations were found in the other socio-demographic variables. In other study about fear of movement age does not differentiate the level of kinesiophobia in patients with multiple sclerosis, Parkinson’s disease and among people after stroke. However, in the case of multiple sclerosis a slight tendency of an increase in the level of kinesiophobia was noticed with the age and duration of the disease in these patients. In the study by Larsson et al. (2016), high levels of kinesiophobia were found among older adults with chronic pain (54). The place of residence did not differentiate kinesiophobia in people with Parkinson’s disease and after a stroke but it differentiated kinesiophobia in people suffering from multiple sclerosis – living in larger cities was a premise for a lower level of kinesiophobia – compared to inhabitants of villages and small towns (30, 38). We did not observe such correlation in the case of women with BC. The level of education and marital status did not differentiate kinesiophobia in people with Parkinson’s disease, multiple sclerosis and after a stroke as well (30).

BC is increasingly a chronic, rather than mortal, disease. It is therefore important to educate patients that it is possible to live actively with cancer. According to Irwin et al. (2004), most of the BC survivors are not still meeting the PA recommendations proposed for the general adult population. Efforts to encourage and facilitate PA among these women would be an important tool to decrease obesity, prevent postdiagnosis weight gain and improve BC prognosis (7). Moreover, greater decreases in PA after diagnosis are observed among women who are treated with radiation and chemotherapy compared with women who undergo surgery only or who are treated with radiation only (10). Kinesiophobia is also common in cancer patients during chemotherapy with totally implantable venous access ports and it is closely related to the subjective experience of daily activities, which requires more attention and early intervention to reduce the potential adverse effects (55).

### 4.1 Study limitations and future lines of the research

The current study is a cross-sectional study – it would be worthwhile to conduct a longitudinal study in the future in order to observe how the attitudes of oncology patients towards PA change during several years of cancer treatment. The aim would be to eliminate the phenomenon of kinesiophobia at every type and stage of oncological treatment. In the future, cancer patients should also be examined during radiotherapy, hormone therapy, immune therapy and chemotherapy because each type of treatment creates different conditions for PA. People suffering from malignancies other than BC should also be examined (PA is important in prostate or colon cancer as well).

## 5 Conclusions

The incentive to remain or return to a normal physical condition is an important motive for PA after BC surgery. Instructions and motivation are important in starting up and/or continuing PA after BC treatment. While in considering the vital benefits of PA for BC patients healthcare professionals, and in particular physiotherapists, must be aware of the need for information and the patients’ motive for exercise and PA to be able to guide and encourage them individually. The professionals must also understand the need of motivation for these women to take control over their lives (56). According to Jochem & Leitzmann (2022), with regard to the source of information on PA cancer survivors prefer oncologists followed by nurses (1). Kinesiophobia might be overcome through integrative and innovative medical healthcare concepts that include PA and the corresponding counselling, guidance and support during all stadiums of cancer treatment. Fear of movement is associated with the perceived global health status of cancer survivors and decreases after rehabilitation (57). Oncologists, physiotherapists, exercise physiologists, and for example diabetologists should collaborate with each other in order to recommend patients doses and types of PA adapted to their age, physical condition and comorbidities, such as diabetes.

## Data availability statement

The raw data supporting the conclusions of this article will be made available by the authors, without undue reservation.

## Ethics statement

The study was conducted in accordance with the Declaration of Helsinki. Our study protocol was reviewed and approved by Bioethics Committee at Poznan University of Medical Sciences (16 June 2021). A decision was issued that the study does not have the features of a medical experiment and that our diagnostic survey may be conducted among cancer patients in the oncological surgery department of breast diseases.

## Author contributions

Conceptualization, E-MM. Methodology, E-MM. Software, AC-B, AM-B, E-MM. Validation, MR, E-MM, PN, ZW, TU, WS, UC, AD, AC-B, AM-B. Formal analysis, AC-B, AM-B, E-MM. Investigation, TU, PN, WS, JW, MR, UC, AD. Resources, E-MM. Data curation, E-MM. Writing—original draft preparation, E-MM. Writing—review and editing, E-MM. Visualization, MR, E-MM. Supervision, E-MM. Project administration, E-MM. All authors contributed to the article and approved the submitted version.
