# Supplementary material for: The level of kinesiophobia in breast cancer women undergoing surgical treatment
Source: Front Oncol. 2023 Feb 2;13:1010315. doi: 10.3389/fonc.2023.1010315 (PMC9932589; doi:10.3389/fonc.2023.1010315)
Supplement: Supplementary file 2 [file DataSheet_2.pdf]

Proszę o zaznaczenie odpowiedzi na następujące pytania:

Zgodnie z rekomendacjami WHO (Światowej Organizacji Zdrowia), osoby dorosłe (zarówno zdrowe jak i chorujące na nowotwór) powinny wykonywać aktywność fizyczną przez minimum 150–300 minut tygodniowo o umiarkowanej intensywności lub 75–150 minut o dużej intensywności.

1. **Czy znała Pani aktualne wymienione powyżej rekomendacje WHO dotyczące zdrowych osób dorosłych? DID YOU KNOW WHO PHYSICAL ACTIVITY RECOMMENDATIONS FOR HEALTHY ADULTS?**

   Tak YES

   Nie NO
2. **Czy zna Pani aktualne wymienione powyżej rekomendacje WHO dotyczące osób dorosłych chorujących na nowotwór? DO YOU KNOW WHO PHYSICAL ACTIVITY RECOMMENDATIONS FOR ADULTS WITH CANCER?**

   Tak YES

   Nie NO
3. **Czy przed zachorowaniem na nowotwór piersi prowadziła Pani aktywny styl życia zgodny z rekomendacjami WHO? DID YOU LEAD AN ACTIVE LIFESTYLE IN ACCORDANCE WITH WHO RECOMMENDATIONS BEFORE DIAGNOSIS?**

   Tak YES

   Nie NO
4. **Od ilu lat jest Pani aktywna fizycznie zgodnie z rekomendacjami WHO? .....**

   FOR HOW MANY YEARS HAVE YOU BEEN PHYSICALLY ACTIVE ACCORDING TO WHO RECOMMENDATIONS?
5. **Od ilu lat NIE jest Pani aktywna fizycznie niezgodnie z rekomendacjami WHO? .....**

   FOR HOW MANY YEARS YOU HAVE NOT BEEN PHYSICALLY ACTIVE (NOT ACCORDING TO WHO RECOMMENDATIONS)?
6. **Czy aktualnie w trakcie leczenia (po diagnozie nowotworu piersi) prowadzi Pani aktywny styl życia zgodny z rekomendacjami WHO?**

   DO YOU LEAD AN ACTIVE LIFESTYLE NOW DURING TREATMENT IN ACCORDANCE WITH WHO REC. ?

   Tak YES

   Nie NO
7. **Czy po zakończeniu leczenia zamierza Pani prowadzić aktywny styl życia zgodny z rekomendacjami WHO?**

   ARE YOU GOING TO LEAD AN (WHO) ACTIVE LIFESTYLE AFTER TREATMENT?

   Tak YES

   Nie NO

   Nie wiem I DONT KNOW
8. **Na jaki rodzaj nowotworu Pani choruje:**

   WHAT KIND OF BREAST CANCER DO YOU SUFFER?

   Podtyp luminalny A TYPE LUMINAL A

   Podtyp luminalny B TYPE LUMINAL B

Podtyp bazalny (potrójnie ujemny) BASAL TYPE(TRIPLE NEGATIVE)

Podtyp HER-2 dodatni TYPE HER+

9. **W jakim stopniu zaawansowania choroby zdiagnozowano u Pani nowotwór piersi?**

   WHAT STAGE OF CANCER HAVE YOU BEEN DIAGNOSED?

   Stopień O

   Stopień 1

   Stopień 2

   Stopień 3

   Stopień 4

10. **Jakie metody leczenia zostały Pani zarekomendowane?**

    WHAT KIND OF TREATMENT DID YOU HAVE RECOMMENDED?

    Operacja chirurgiczna SURGICAL

    Radioterapia RADIOTHERAPY

    Chemioterapia CHEMOTHERAPY

    Hormonoterapia HORMONOTHERAPY

11. **Czy czuje Pani obecnie zmęczenie?**

    DO YOU FEEL FATIGUE NOW?

    Nie NOT

    Lekkie LIGHT

    Średnie AVERAGE

    Silne STRONG

12. **Czy odczuwa Pani obecnie dolegliwości bólowe?**

    DO YOU FEEL PAIN NOW?

    Nie NOT

    Lekkie LIGHT

    Średnie AVERAGE

    Silne STRONG

13. **Jak ocenia Pani obecnie swoją kondycję fizyczną?**

    HOW DO YOU RATE YOUR PHYSICAL CONDITION?

    Kiepsko QUITE BAD

    Średnio AVERAGE

    Dobrze QUITE GOOD

14. **Jak ocenia Pani obecnie swoją kondycję psychiczną?**

    HOW DO YOU RATE YOUR MENTAL CONDITION?

    Kiepsko QUITE BAD

    Średnio AVERAGE

    Dobrze QUITE GOOD

15. **Masa (kg): WEIGHT**
16. **Wysokość ciała (cm): HEIGHT**

17. **Na jakie choroby przewlekłe oprócz nowotworu Pani choruje? OTHER CHRONIC DISEASES**

Nie choruję na żadne dodatkowe choroby przewlekłe I DONT HAVE

Cukrzyca DIABETES

Nadciśnienie HYPERTENSION

Otyłość OBESITY

Miażdżyca ATHEROSCLEROSIS

Osteoporoza OSTEOPOROSIS

Choruję na inne dodatkowe choroby przewlekłe OTHER CHRONIC DISEASES

18. **Jak ciężko przeszła Pani zakażenie koronawirusem?**

HOW HAVE YOU PASSED COVID-19 INFECTION?

Nie przechodziłam zakażenia koronawirusem I DID NOT PASS

Nie wiem czy przechodziłam zakażenie koronawirusem I DONT KNOW IF I PASS

Przechodziłam zakażenie koronawirusem w stopniu lekkim I PASS LIGHTLY

Przechodziłam zakażenie koronawirusem w stopniu umiarkowanym AVERAGE

Przechodziłam zakażenie koronawirusem w stopniu ciężkim I PASS STRONGLY

I. Dane społeczno-demograficzne: SOCIO-DEMOGRAPH.

1. Osiągnięte wykształcenie: EDUCATION

Podstawowe PRIMARY

Średnie SECONDARY

Wyższe HIGHER

2. Miejsce zamieszkania: PLACE OF RESIDENCE

Wieś VILLAGE

Małe miasto (do 20 tys. mieszkańców) SMALL CITY 20 000

Średnie miasto (20-100 tys. mieszk.) AVERAGE CITY 20-100 000 INHABITANTS

Duże miasto (powyżej 100 tys. mieszk.) LARGE CITY MORE THAN 100 000

3. Stan cywilny: ACTUAL MARITAL STATUS

Singielka SINGLE

W związku lub małżeństwie MARRIAGE/IN RELATIONSHIP

Rozwiedziona lub wdowa WIDOW / DIVORCED

4. Aktualna sytuacja zawodowa: ACTUAL OCCUPATIONAL STATUS

Osoba aktywna zawodowo ACTIVE

Studentka STUDENT

Emerytka PENSIONER

Rencistka PENSIONER (HEALTHY REASONS)

Osoba bezrobotna UNEMPLOYED

5. Wiek: AGE

+ TAMPA SCALE OF KINESIOPHOBIA QUESTIONS
